# Exploring the Impact of Cerebral Microbleeds on Stroke Management

**DOI:** 10.3390/neurolint15010014

**Published:** 2023-02-01

**Authors:** Anastasia Sousanidou, Dimitrios Tsiptsios, Foteini Christidi, Stella Karatzetzou, Christos Kokkotis, Aimilios Gkantzios, Chrisostomos Bairaktaris, Vaia Karapepera, Paschalina Bebeletsi, Ioanna Karagiannakidou, Marinos Marinidis, Nikolaos Aggelousis, Konstantinos Vadikolias

**Affiliations:** 1 Neurology Department, Democritus University of Thrace, 68100 Alexandroupolis, Greece; 2 Department of Physical Education and Sport Science, Democritus University of Thrace, 69100 Komotini, Greece

**Keywords:** cerebral microbleeds, stroke, thrombolysis, dementia, depression, prognosis

## Abstract

Stroke constitutes a major cause of functional disability and mortality, with increasing prevalence. Thus, the timely and accurate prognosis of stroke outcomes based on clinical or radiological markers is vital for both physicians and stroke survivors. Among radiological markers, cerebral microbleeds (CMBs) constitute markers of blood leakage from pathologically fragile small vessels. In the present review, we evaluated whether CMBs affect ischemic and hemorrhagic stroke outcomes and explored the fundamental question of whether CMBs may shift the risk–benefit balance away from reperfusion therapy or antithrombotic use in acute ischemic stroke patients. A literature review of two databases (MEDLINE and Scopus) was conducted to identify all the relevant studies published between 1 January 2012 and 9 November 2022. Only full-text articles published in the English language were included. Forty-one articles were traced and included in the present review. Our findings highlight the utility of CMB assessments, not only in the prognostication of hemorrhagic complications of reperfusion therapy, but also in forecasting hemorrhagic and ischemic stroke patients’ functional outcomes, thus indicating that a biomarker-based approach may aid in the provision of counseling for patients and families, improve the selection of more appropriate medical therapies, and contribute to a more accurate choice of patients for reperfusion therapy.

## 1. Introduction

Stroke not only accounts for the majority of acquired disability cases in adults but also remains the second leading cause of death, despite the significant progress achieved in preventive measures and therapeutic interventions [1,2]. Given the age-related nature of the disease, since more than half of all patients are aged over 65 [3], in combination with the increasing global population and the constant improvement of life expectancy [4], the number of stroke survivors will rise in an unprecedented manner. Consequently, the early and precise recognition of patients with unfavorable prognoses is essential in order to personalize treatment and tailor rehabilitation to each individual’s needs [5].

The problem of prompt poststroke outcome forecasting has proven to be puzzling due to the disease’s heterogeneity in terms of its etiology and pathophysiology, thus sparking the proliferation of publications exploring the prognostic potential of stroke biomarkers [6,7]. Several stroke prognosis scales have been developed [8], including the National Institutes of Health Stroke Scale (NIHSS) [9], and various neurophysiological techniques have been utilized [10] to provide timely information of each patient’s recovery potential, thus aiding physicians in developing appropriate stroke care plans.

Until now, the ability of several biomarkers to offer valid information on prospective stroke patients’ functional outcomes have been investigated [11,12], with cerebral microbleeds (CMBs) among them. The term CMBs is used to describe the radiological observation of tiny perivascular hemorrhages, usually seen in elderly individuals, appearing as hypointense, rounded lesions (usually 2–10 mm in diameter) on T2*-weighed gradient-recalled echo (T2*-GRE) and related MRI sequences that are sensitive to magnetic susceptibility [13]. These lesions, originally coined ‘hemorrhagic lacunes’, were first described in 1994 by Scharf et al. in patients with hypertensive cerebrovascular disease and intracerebral hemorrhage (ICH) associated with ischemic white matter disease and lacunar infarcts [14]. Later, in 1996, Offenbacher et al. further characterized them using T2*-GRE MRI sequences, leading to the current definition of ‘microbleeds’ [15].

As far as the epidemiological profile of CMBs is concerned, age [16,17] appears to be the most decisive risk factor, since their prevalence increases with age [18], and a striking exacerbation after the age of 75 has been observed [19]. Additionally, male sex [19], systemic hypertension [16,17,20,21], and heavy smoking [20,21] have been linked with CMB development. However, no independent relationships were found with other cardiovascular risk factors [19].

Although the exact mechanism of the formation of the lesions remains undiscovered, the development of these abnormalities is considered to be of hemorrhagic origin. The available histopathological studies, although limited in number [22,23,24,25,26], suggest that CMBs are composed of small collections of blood-breakdown products comprised within perivascular macrophages. This direct neuroimaging–pathological correlation was first reported by Fazekas et al., who, after studying 11 autopsied brains, concluded that the hypointense lesions on T2*-GRE MRI corresponded to focal accumulations of macrophages containing hemosiderin close to small abnormal vessels, stemming from either hypertensive angiopathy or cerebral amyloid angiopathy (CAA) [22]. Furthermore, the microangiopathic disorder that is responsible for the concept of CMBs seems to affect their topography, as CMBs of hypertensive etiology typically appear in the basal ganglia, thalamus, brainstem, and cerebellum, whereas CAA is characterized by a lobar, cortical–subcortical distribution [13].

Along with leukoaraiosis (LA), CMBs constitute an imaging marker of cerebral small vessel disease (SVD), a degenerative vascular disease most commonly observed among elderly individuals [27,28,29], but they are unique among the MRI manifestations of SVD, as they offer clear evidence of microvascular leakiness, causing blood products, such as inflammatory cells and various plasma proteins, to extravasate through the vessel wall, leading to direct structural damage to the surrounding tissue. Furthermore, the high concentration of hemosiderin in these tiny bleeds can potentially disrupt cortical electrical activity [30,31] or disturb the function of neurons located near the microbleeds [32].

Thus, although CMBs were previously considered an incidental imaging finding with no major implications for clinical practice, accumulated evidence suggests that the CMB burden is coupled with a group of specific clinical manifestations, including balance and gait impairments with subsequent falls, disability, and cognitive decline. Studies recruiting patients with vascular cognitive impairment [33], Alzheimer’s disease [34], and normal individuals [35] have suggested a possible strong impact of CMBs on cognition and CMBs in conjunction with lacunar infarcts and white matter hyperintensities (WMHs) are considered to be the major underlying pathology in cases of vascular dementia [36]. Specifically for Alzheimer’s disease, patients with multiple CMBs performed worse on the Mini-Mental State Examination (MMSE) despite having a similar disease duration and had lower cerebrospinal fluid amyloid beta 1-42 levels than those without CMBs [34], but no significant relationship between CMBs and APOE allele status has been proven [19]. Regarding gait disturbances, a higher number of CMBs was associated with a shorter stride length and poorer performance on the Tinetti and Timed-Up-and-Go tests in nondemented elderly individuals. These relations seemed to be explained by CMB location in the temporal lobe, the frontal lobe, and the basal ganglia, including the thalamus, and emerged independently of other coexisting markers of SVD [37].

Several assessment tools have been utilized to quantify the presence and severity of CMBs and MRI brain scans, since the evaluation of the presence, number, size, and distribution of cerebral microbleeds can be of utmost importance in monitoring underlying diseases and implementing risk-modifying measures. The most commonly used approach is the Microbleed Anatomic Rating Scale (MARS) score, which assesses the presence and number of microbleeds in each anatomical region and individual cerebral lobes [38,39], allowing the investigation of regional correlations among white-matter changes, microbleeds, and clinical factors. Furthermore, the rating form includes a convenient summary of the total microbleed counts for the whole brain and each anatomical region, as well as a clear guide to the anatomical boundaries of the cerebral lobes and regions [38]. Additionally, LA, visible as white matter hyperintensity on MRI, seems to share a common development pathway with CMB. Interestingly, the presence of CMBs was found to show parallels with leukoaraiosis severity, with both being strongly associated with the clinical manifestation of cerebrovascular symptoms [20,40,41].

Since the presence of CMBs denotes the occurrence of blood leakage from pathologically fragile small vessels, a fundamental question may be raised regarding whether CMBs may shift the risk–benefit balance away from antithrombotic use in some patients. Another relevant clinical dilemma is whether CMBs increase the risk of intracerebral hemorrhage (ICH) after intravenous thrombolysis (IVT) or another type of reperfusion therapy for patients with acute ischemic stroke (AIS). Taking into consideration the clinical relevance and the potential prognostic role of CMBs within an aging population, as well as the emerging need for accurate forecasting of each stroke individual’s propensity for recovery, the purpose of the present study was to review all available literature published within the last decade dealing with CMBs as outcome predictors not only after reperfusion therapy for AIS but also in hemorrhagic and AIS survivors who had not undergone reperfusion therapy.

## 2. Materials and Methods 

The Preferred Reporting Items for Systematic Reviews and Meta-Analyses (PRISMA registration number: CRD42023388876) were used to guide this study. Our study’s methods were designed a priori.

### 2.1. Search Strategy

Two investigators conducted literature reviews using two databases (MEDLINE and Scopus) (AS and DT) to trace all relevant studies published between 1 January 2012 and 9 November 2022. The search terms were as follows: (“cerebral microbleeds” OR “cortical microbleeds”) AND (“stroke prognosis” OR “stroke recovery” OR “intravenous thrombolysis” OR “mechanical thrombectomy”). The retrieved articles were also hand-searched for any further potential eligible articles. Any disagreement regarding the screening or selection process was solved by a third investigator (KV) until a consensus was reached.

### 2.2. Selection Criteria

Only full-text original articles published in the English language were included. Secondary analyses, reviews, guidelines, meeting summaries, comments, unpublished abstracts, and studies conducted on animals were excluded. There was no restriction on study design or sample characteristics.

### 2.3. Data Extraction

Data extraction was performed using a predefined data form created in Excel. We recorded the type of stroke, authors, year of publication, type of study, number of participants and their age (including the mean/median, standard deviation, and range when available), gender, level of education, body mass index (BMI), cerebrovascular risk factors, medication, previous stroke, follow-up time, method of microbleed assessment, time of MRI test execution, the scale used to assess stroke severity and clinical outcome, the main results, and also the time of recanalization for the studies in the reperfusion group.

### 2.4. Data Analysis

No statistical analysis or meta-analysis was performed due to the high heterogeneity observed among studies. Thus, the data were only descriptively analyzed.

## 3. Results

### 3.1. Database Searches

**Figure 1:**
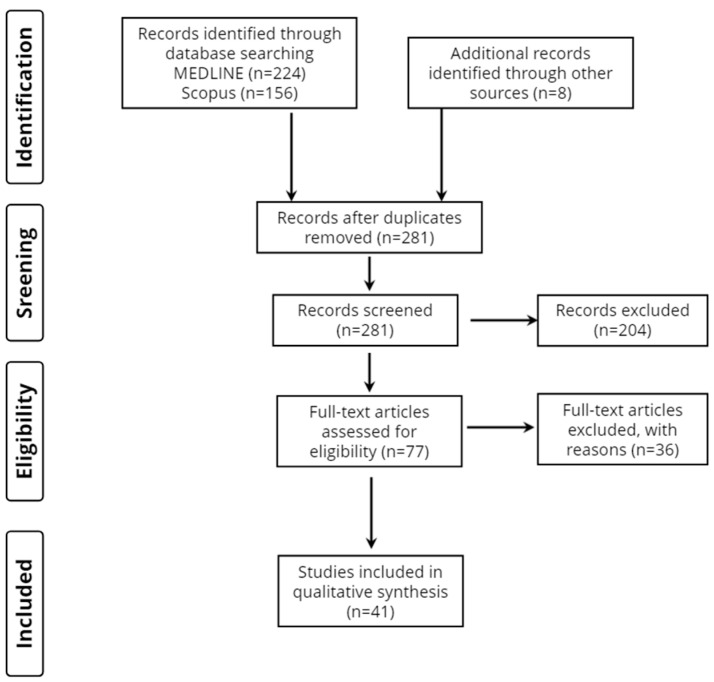
Study flow diagram (PRISMA flowchart).

Overall, 388 records were retrieved from the database search. Duplicates and irrelevant studies were excluded; hence, a total of 77 articles were selected. After screening the full texts of the articles, 41 studies were eligible for inclusion (Figure 1).

### 3.2. Study Characteristics

Forty-one publications fulfilled our inclusion criteria. Seventeen studies focused entirely on AIS, three included patients with either AIS or transient ischemic attack (TIA), one had only patients with TIA, five studies reported only on ICH patients, one enrolled only patients with subarachnoid hemorrhage (SAH), ten enrolled patients who received IVT, two included participants undergoing mechanical thrombectomy (MT), and two of them studied patients regardless of their reperfusion therapy type. With regard to the origins of the studies, twenty-two were from Asia, twelve came from Europe, six were from America, and one was from Australia (Table 1).

### 3.3. Stroke Patient Groups and Demographic Profile

The total number of stroke patients included in all studies ranged from *n* = 39 [82] to *n* = 2002 [75]. Across the 41 studies, 3 studies had a disease sample size between 1 and 100 patients, 9 studies between 101 and 200, 5 studies between 201 and 300, 8 studies between 301 and 400, 3 studies between 401 and 500, and 13 studies had a disease sample size larger than 500 patients. The mean/median patient age ranged from 54.08 years [78] to 78.20 years [68].

### 3.4. Reference Groups

In none of the 41 included studies were stroke patients contrasted with demographically matched healthy individuals and none of the studies included a disease-control group other than stroke patients.

### 3.5. Time of MRI Execution

In 2 studies, MRI testing was performed at baseline, in 10 it was performed on admission, in 4 within 24 h of symptom onset, in 1 study MRI was executed within 3 days from symptom onset, in 1 study within 5 days, in 3 studies within 7 days of symptom onset, in 1 study in the first 14 days, in 1 within the first 3 months, in 6 studies before reperfusion therapy, and in 2 studies MRI was performed either before or within 24 h of reperfusion treatment.

### 3.6. Method of Cerebral Microbleed Assessment

Regarding the method of CMB neuroimaging assessment, ten studies preferred the MARS method, two preferred the Brain Observer Microbleed Scale (BOMBS), one assessed CMBs according to the Microbleed Study Group (MSG) method, twenty-two counted the number of CMBs, sixteen noted their topographical distribution, four reported on CMB presence, and finally, one study assessed their size.

### 3.7. Scales of Stroke Severity and Prognosis/Clinical Outcome

The NIHSS and modified ranking scale (mRS) were used simultaneously in 19 studies, NIHSS alone was used in 6 studies, and mRS was utilized exclusively in 4 studies. In the rest of the studies, a combination of scales of stroke severity and clinical outcome was used. More specifically, in one study, NIHSS was combined with the Mini-Mental State Examination (MMSE), Geriatric Depression Scale (GDS), and Barthel Index (BI); in another it was combined with mRS, MMSE, the Luben Social Network Scale (LSNS), and GDS, and in another it was combined with mRS and the Glasgow Coma Scale (GCS). Moreover, in one study, mRS was combined with GCS, in another with the Telephone Interview for Cognitive Status (TICS), and in another with either TICS or the Informant Questionnaire on Cognitive Decline in the Elderly (IQCODE). Finally, in one study, the executive function of patients was assessed with the Stroop test, Word Fluency, Trail Making Test Part B, Weigl Color Form Sorting Task, and the Modified Card Sorting Test.

## 4. Discussion

A review of the literature published over the last decade was conducted to elucidate the prognostic value of CMBs after stroke and their role in regard to treatment outcomes. Forty-one original full-text articles dealing with the potential benefit of the assessment of pre-existing CMBs in stroke prognosis were identified and divided into three groups.

### 4.1. Reperfusion Therapy

#### 4.1.1. Intravenous Thrombolysis

The most critical complication of reperfusion therapy in stroke patients is symptomatic intracerebral hemorrhage (sICH) and the timely identification of high-risk patients could potentially limit its devastating effects. Since CMBs have been previously linked to ICH, Zand et al. [42] pondered whether the presence of CMBs increased the risk of sICH in AIS patients receiving IVT. In their analysis, sICH frequencies did not differ between patients with or without CMBs, and no deviation occurred when the locations of CMBs, whether strictly lobar or deep/infratentorial, were considered. However, patients with >10 CMBs were more likely to experience sICH during hospitalization and the correlation remained significant even after adjusting for age, gender, hypertension, diabetes mellitus, hyperlipidemia, history of stroke, atrial fibrillation, tobacco use, admission to NIHSS, and field strength of the MRI scan. Regarding mortality, there was no difference in the rate of in-hospital death of patients with or without CMBs and even though there was a correlation between in-hospital mortality and >10 CMBs, the relationship failed to reach statistical significance. Moreover, the authors noticed that the majority of sICHs stemmed from pre-existing CMBs, supporting the hypothesis that CMB presence reflects severe microangiopathy. Taking all of the aforementioned findings into consideration, the authors argued in favor of early MRI acquisition in patients receiving IVT, since this could provide crucial prognostic information for sICH and help clinicians take prompt action, as patients with a high CMB burden could benefit post-IVT from close monitoring, more restricted blood pressure control, or perhaps a lower blood pressure target.

Nagaraja et al. [43] also assessed the potential risk factors for hemorrhagic transformation (HT) after IVT. In their study, participants experiencing HT tended to be older, non-white, had atrial fibrillation, higher baseline NIHSS scores, lower cholesterol and triglyceride levels, CMBs, and non-lacunar infarcts. In particular, the presence of CMBs was an independent predictor of HT, and the only other factor that was able to forecast HT independently of CMBs was the baseline NIHSS score. Patients with HT had a decreased chance of discharge to home, but the presence of CMBs did not increase their risk of in-hospital death or discharge to a rehabilitation clinic or hospice. The location of CMBs, either in lobar or deep structures, did not alter the risk of HT; however, a correlation between lobar CMBs and parenchymal hemorrhage (PH) was noted. On the other hand, neither the severity nor the location of LA seemed to be able to predict HT and CMBs had no influence on HT in the presence of LA, indicating that the association of LA with poor functional outcomes was probably not mediated by HT or sICH. Since the question of whether the appearance of HT due to CMBs outweighs the benefits of IVT remained unanswered, the authors did not suggest withholding rt-PA treatment for AIS patients with a high CMB burden.

Similarly, Schlem et al. [44] compared AIS patients receiving alteplase or a placebo and recorded their functional outcomes and sICH rates. The presence of CMBs had no impact on 90-day excellent functional outcomes or death, nor did it affect HT occurrence, and although sICH was more common in patients with CMBs, the relationship failed to reach statistical significance. Patients receiving IVT treatment had significantly lower mRS scores compared to placebo patients, without any proof of heterogeneity regarding the presence, number, or topographical distribution of CMBs. Considering that no evidence of CMB burden negating the beneficial effects of IVT was found, the authors suggested that clinicians should not withhold alteplase treatment from eligible patients. However, they also admitted that the sample size of patients with >10 CMBs was too small to study whether a higher number of CMBs diminished the likelihood of favorable IVT outcomes.

Likewise, Chacon-Portillo et al. [45] dealt with the potential of pre-existing CMBs to cause HT, either symptomatic or asymptomatic, in AIS survivors treated with IVT. Neither the presence nor the location of CMBs had any impact on ICH risk, but patients with >10 CMBs were at an increased risk of sICH. For HT, only higher baseline NIHSS scores, lower platelet counts, and large vessel disease were independent predictors. Futhermore, CMBs did not alter 90-day functional outcomes. Taking into account that the number of patients with >10 CMBs and the rate of HT was very low, the authors proposed that delaying AIS treatment to establish the CMB burden is unnecessary, and even when T2*-GRE MRI sequences are utilized, physicians should not be deterred from treating patients with alteplase based on CMB status alone.

In addition, AIS patients receiving IVT and undergoing susceptibility-weighted imaging before therapy were recruited by Yan et al. [46] in order to investigate the influence of CMBs on functional outcomes and the various HT subtypes. The presence of CMBs did not raise the risk of HT 24 h after IVT or of poor functional outcomes, but patients with ≥3 CMBs had a significantly increased risks of parenchymal hemorrhage (PH) and poor functional outcomes, and this correlation was significant even when adjusting for LA volume. Moreover, a high grade of CMB burden showed a possible correlation with ICH. Finally, the presence of CMBs was correlated with a higher risk of extra-ischemic ICH. The authors proposed that the link between CMBs with PH could potentially be explained by the possibility that cerebral microbleeds are a sign of impaired blood–brain barrier function and increased vascular vulnerability. Still, further studies on IVT utilization in patients with increasing CMB burden are needed to aid decision-making, given the superior outcomes of alteplase-treated patients and since the strong effect of the time from symptom onset to treatment on stroke outcomes cannot be ignored, performing pre-thrombolysis MRI to detect CMBs is hard to justify.

To evaluate whether the radiological markers of SVD could be independent predictors of hemorrhagic complications and thus constitute a possible contraindication for IVT, Capuana et al. [47] enrolled AIS patients receiving alteplase and recorded their CMB and LA status. The presence of CMBs was independently associated with PH and ICH, especially with severe ICH, but CMB burden did not influence functional outcomes or 1-week and 90-day mortality. Regarding the topographical distribution of CMBs, deep or infratentorial locations resulted more frequently in any form of ICH, whereas a lobar location resulted more frequently in remote PH. Regarding LA, a higher burden of WMHs was only related to more severe ICH and did not influence functional outcomes or mortality. Taking all these findings into consideration, the authors suggested that the decision regarding IVT administration should be personalized to each patient and cannot be based exclusively on radiological signs.

Moreover, Turc et al. [48] recruited AIS patients receiving only alteplase and recorded their outcomes. The presence of CMBs upon pretreatment MRI increased the risk of a higher mRS scores at 3 months, but after adjustment for age, hypertension, and atrial fibrillation, the relationship did not remain statistically significant. This was also true when the CMB location or presumed underlying vasculopathy was considered. Additionally, no correlations between CMB burden, location, or presumed underlying vasculopathy and sICH occurrence were found. Given the conflicting results of the studies found in the literature, the authors call for an individual patient data meta-analysis to determine whether there exists a subgroup of patients with CMBs which seems to have an independent risk of poor outcomes after 3 months that is so great that it might outweigh the expected benefit of reperfusion therapy and thus justify the widespread use of MRI in an acute ischemic stroke setting.

In their cohort, Dannenberg et al. [49] revealed that in patients with 2–4 CMBs and ≥5 CMBs, the occurrence of sICH and PH was more common than in those without CMBs, but a single CMB did not alter hemorrhagic risk. The graded relationship of the risk of hemorrhagic complications with increasing baseline CMB number remained significant after adjustments for age, LA grade, atrial fibrillation, onset-to-treatment time, prior statin use, and systolic blood pressure on admission. Notably, prior statin use was also independently associated with PH. Additionally, having ≥5 CMBs was weakly correlated with an unfavorable 3-month outcome, and hence no conclusion on the effectiveness of IVT in patients with multiple CMBs could be drawn and no clear CMB threshold has been identified below which no benefit or harm of IVT could be observed.

Since the underlying mechanism of remote intracerebral hemorrhage (r-ICH) remains unknown in most cases, Drelon et al. [50] tested the hypothesis that r-ICHs occurring in AIS patients receiving alteplase stemmed from pre-existing brain lesions, being either hemorrhagic transformations of a silent brain infarct or bleeding from local lesions such as CMBs. Although patients experiencing r-ICHs had more frequently strictly lobar CMBs, suggesting amyloid angiopathy, and ≥5 CMBs, in the majority of the cases the r-ICH occurred in an area that appeared normal upon pre-treatment MRI. Thus, the prognosis of r-ICH was more accurate when based on clinical characteristics, such as age and baseline systolic blood pressure, than on imaging variables. Moreover, the rate of r-ICH was low and the majority of cases were asymptomatic, indicating that a momentary CMB burden does not constitute an absolute exclusion criterion for IVT. The authors concluded that further studies focusing on long-term outcomes, especially for epileptic seizures and cognitive decline, are needed.

Finally, Prats-Sánchez et al. [51] studied the potential risk factors for rPH in AIS patients receiving thrombolysis. Markers of SVD, such as CMBs, cortical superficial siderosis (CSS), LA, and recent silent ischemia (RSI), were recorded and a follow-up CT was performed after treatment to detect any extraischemic hemorrhages. Based on this procedure, a strictly lobar location and the presence of multiple CMBs, as well as the presence of CSS and RSI, were more common in patients experiencing rPH, but only a lobar distribution of CMBs, indicative of CAA-related vasculopathy, and RSI were independently associated with rPH. The underlying mechanism causing vessel rupture after amyloid deposition remains unclear, but it has been hypothesized that vascular amyloid-β deposition makes small cortical vessels brittle and fragile, increasing their vulnerability to bleeding when hemostasis is acutely impaired by thrombolysis. As expected, patients with hemorrhagic complications had a decreased rate of favorable outcomes at 3 months and survival, indicating that extra attention should be given to this uncommon but potentially lethal event.

#### 4.1.2. Mechanical Thrombectomy

To analyze the complications of MT, Shi et al. [52] enrolled patients with large-vessel-occlusion strokes and recorded potential imaging markers of deterioration upon pre-treatment MRI and the clinical outcomes of participants until discharge. Neither HT or PH occurrence, procedure-related vessel perforation, in-hospital mortality, or unfavorable outcomes were more common in patients with CMBs compared to those without them. This remained true even when topographical distribution and the number of CMBs were taken into account. Moreover, the successful revascularization rates did not differ between patients with and without CMBs. On the contrary, WMHs alone significantly raised the risk of hemorrhagic complications and death before discharge, but this was not observed in patients with confluent CMBs and WMHs or patients with ≥2 CMBs. Based on the above, the presence of CMBs could not represent a MT contraindication, but since there was only one patient with ≥5 CMBs, the authors admitted that the effect of multiple CMBs on stroke patients undergoing MT could not be studied.

Lee et al. [53] also tested the effect of CMBs on the clinical outcomes of AIS stroke patients undergoing MT and estimated the influence that HT, WMH, and procedural success could have on it. For this purpose, patients were trichotomized into subgroups of 0, 1–4, or ≥5 CMBs, and the rates of hemorrhagic complications, successful reperfusion, and poor unfavorable outcomes were recorded. Participants with an increasing burden of CMBs had more hemorrhagic complications, and PH in particular, as well as lower rates of reperfusion success and significantly higher mRS scores. Although the direct effect of CMBs on functional outcomes was statistically significant, the effect of CMBs on poor functional status was partially mediated by WMHs, hemorrhagic transformation, and lower rates of successful reperfusion, in order of their percentage of the mediation size. These data suggest that the negative influence of CMBs on functional outcomes is not fully explained by hemorrhagic complications, suggesting that there might be additional pathophysiological pathways. If true, the association of CMBs with poor functional outcomes could be generalized to stroke patients beyond reperfusion therapy, but research on this matter is scarce.

#### 4.1.3. Any Form of Reperfusion Therapy

Choi et al. [54] studied the impact of CMBs on the clinical outcomes and hemorrhagic complications of AIS patients undergoing revascularization therapies. The functional outcomes did not differ between participants with or without CMBs when the entire cohort was considered. However, in patients achieving recanalization, favorable outcomes were significantly more frequent among patients with no CMBs. In particular, the presence of ≥5 CMBs and a lobar distribution was correlated with poor functional outcomes, and in patients achieving complete reperfusion, the ones without CMBs had lower mRS scores. This difference was further amplified when patients were treated with MT rather than solely with IVT. On the contrary, the risk for HT and sICH was significantly increased with the presence of CMBs for all patients, regardless of treatment success. Neither the mortality rate, new CMB development, or the frequency of early neurological deterioration were statistically different between the two groups. Based on the fact that patients achieving recanalization had significantly better functional outcomes, the authors suggested that IVT and MT should not be withheld from eligible patients based on the CMB burden alone. Lastly, to illuminate the potential influence of CMBs on stroke survivors, Gratz et al. [55] conducted a study on AIS patients treated with either IVT, endovascular therapy, or IVT followed by endovascular therapy and recorded their outcomes. Regarding hemorrhagic complications, no correlation between CMB burden, location, or presumed pathogenesis and the risk of symptomatic or asymptomatic ICH was found, but a positive correlation between CMBs and sICH was reported in the subgroup of patients treated with IVT only. In addition, the CMB number had no impact on the survival rate or 3-month functional outcomes of patients. Only a higher CMB burden slightly increased the risk of ICH outside of the infarct; hence, no suggestion against reperfusion therapy based on the presence of CMBs was made.

In conclusion, the findings of the aforementioned studies dealing with AIS reperfusion therapies should be analyzed with caution. Although recanalization procedures have been established to yield better functional outcomes for AIS survivors, their hemorrhagic complications can result in devastating consequences. Despite the contradictory reports and the lack of consensus in the literature, patients should not be deprived of this potentially life-saving treatment based solely on the presence of CMBs, even though they might increase the early bleeding risk. Further high-quality data from large, prospective cohorts of patients with different grades of CMBs are essential in order to guide physicians in their everyday clinical practice.

### 4.2. Acute Ischemic Stroke/Transient Ischemic Attack

Despite the lengthy literature regarding thrombolysis patients, the prognostic implications of CMBs for AIS survivors not undergoing reperfusion therapy have not been fully investigated. In addition, patients who died before discharge are often excluded from several studies, leading to an underappreciation of stroke-related early mortality. Thus, Zand et al. [57] sought to investigate whether the burden of CMBs could aid the prognostication of in-hospital death. For this purpose, they recruited AIS patients who did not receive IVT and studied their outcomes in combination with the presence of CMBs on MRI. Although the presence of CMBs demonstrated no ability to predict in-hospital death, an independent association between the presence of >4 CMBs and the risk of mortality before discharge was established. Similarly, older age, higher NIHSS, and a history of atrial fibrillation were associated with a greater likelihood of in-hospital death. Since endothelial dysfunction is the proposed underlying pathophysiology of CMBs, the authors further analyzed whether a correlation between CMBs and symptomatic hemorrhagic transformation (sHT) could explain the higher death rate among these patients. Indeed, the rate of sHT was significantly higher among patients with ≥4 CBMs compared to patients with no CMBs, but the number of patients with sHT was too small for the authors to draw reliable conclusions.

#### 4.2.1. Hemorrhagic Transformation

Although HT is a critical event in the acute stage of ischemic stroke and its prognosis could potentially modify the course of treatment, until recently most studies have focused on clinical risk factors, such as blood pressure, blood glucose level, or blood platelet count. Consequently, Liu et al. [58] aimed to identify its radiological predictive signs. Studying multimodal MRI scans (anatomical, diffusion-weighted, and susceptibility-weighted images) of acute cerebral infarction patients, they observed that the infarct size, the presence of CMBs, the relative apparent diffusion (rAD), and the presence of venous anomalies were significantly related to HT, but among these imaging markers only CMBs, rAD, and venous anomalies were independent risk factors for HT. Concerning venous anomalies, the authors proposed that they reflected the severity of hypoxia and the progress of cell anaerobic metabolism. The accumulation of lactic acid substrates further damages the vessel endothelium and leads to vein widening in the compensatory stage. In the decompensation stage, the blood–brain barrier is damaged, permitting HT occurrence after collateral circulation is established. Similarly, the presence of CMBs was a marker of diffuse hemorrhage-prone vasculopathy, possibly contributing to HT pathophysiology.

On the subject of potential HT occurring more often when preexisting CMBs were present, Takahashi et al. [19] enrolled AIS patients who were receiving antithrombotic therapy. They noted no difference in HT frequency, regardless of whether the patients had CMBs or not. Moreover, they categorized patients according to the type of therapy they underwent and found that neither in the subgroup treated with antiplatelets, nor in the subgroup with anticoagulants, did the prevalence of HT show any variance. Hence, the appearance of HT soon after antithrombotic therapy can be better estimated based on the severity of neurological deficits and the volume of ischemic tissue upon admission, rather than the presence of microbleeds upon pretreatment MRI.

#### 4.2.2. Short- and Long-Term Outcomes

Regarding short-term AIS outcomes, Ryu et al. [63] reported on the cumulative effect of SVD on 3-month functional outcomes following ischemic stroke. After calculating the total SVD scores—which included lacunae, CMBs, WMHs, and an enlarged perivascular space—and recording the patients’ mRS scores at 3 months, they noted that a total SVD score of three or four led to a doubled chance of a poor functional outcome, compared with a total SVD score of zero. Moreover, they studied each SVD subtype alone and demonstrated that lacunae, WMHs, and CMBs were independently associated with higher 3-month mRS scores. Nonetheless, the impact that each marker had on functional outcomes was smaller than that of the total SVD score, calling into question the true clinical significance of their separate assessment. Hence, considering that the radiological markers of SVD usually coexist, the utilization of total SVD scores in an AIS setting could better inform physicians about patients’ outcomes. Further investigations of prognoses related to short-term functional outcomes were conducted by Sakuta et al. [64], who enrolled patients with non-cardiogenic minor ischemic stroke, defined as having a baseline NIHSS score < 4, who were treated with antiplatelet therapy and recorded their mRS scores at day 90. A higher count of CMBs was linearly correlated with a higher probability of poor functional outcomes. The CMB number was an independent predictor of poor outcomes but deep WMHs were not, and patients with a higher burden of both CMBs and deep WMHs exhibited a higher risk of poor functional outcomes. Despite both CMBs and WMHs being key SVD factors, the difference in their ability to forecast stroke recovery could be explained by two reasons. First, CMBs usually form when SVD advances to some extent, and second, they are more closely related to brain aging; thus, there is a more strong association of CMBs with these outcomes.

Regarding short- and long-term outcomes, Kim et al. [65] reported on the functional statuses of AIS patients at discharge and after 6 months and their relation with the presence of CMBs. They concluded that the presence of CMBs was associated with higher mRS scores for both periods studied, but only infratentorial CMBs were independently correlated with poor outcomes. Furthermore, the number of CMBs was higher in the poor-functional-outcome group and was positively correlated with increasing WMH scores. These results could be explained by the fact that CMBs are a mirror of underlying brain vascular fragility and, as such, they hinder the patient’s ability to compensate as functionally and as efficiently as those without pre-existing lesions. Secondly, CMBs may reflect focal damage to the brain tissue that, when located in areas important for motor regulatory functions, could interfere with recovery from functional deficits after stroke.

Recently, active cancer has been established not only as a major risk factor for ischemic stroke but also as a marker of poor prognosis, since strokes in patients with cancer are more severe and recur frequently. With the constant advances in cancer therapy, the life expectancy of patients has been consistently increasing, leading to increased interest in improving patients’ quality of life. Hence, Nam et al. [62] tried to elucidate the association between SVD and the prognoses of cryptogenic stroke patients with active cancer at two points in time. They discovered that among the SVD subtypes, only silent brain infarct (SBI) was associated with early neurological deterioration (END), defined as a ≥2-point increase in the patient’s total NIHSS score or a ≥1-point increase in their motor NIHSS score within the first 72 h. Notably, the frequency of END increased proportionally with an increase in the number of SBI lesions. However, neither WMH, CMB, or SBI showed any statistically significant relationship with 3-month unfavorable outcomes. Taking the above into consideration, the authors concluded that although SVD reflects hypoxia and endothelial dysfunction, creating a vulnerable brain environment that is unable to tolerate invasive brain damages such as stroke, special mechanisms related to SBI may be involved in its ability to forecast END. Classifying the high-risk group of patients using SBI and providing appropriate treatment during the acute period may prevent END and minimize disability.

#### 4.2.3. Chronic Outcomes

Regarding the chronic prognosis of AIS, Lau et al. [70], documented the CMB numbers and locations in Chinese stroke patients and followed them up to record their outcomes. The presence of CMBs increased the risk of ICH. Specifically, having ≥5 microbleeds predicted ICH independently of age, sex, vascular risk factors, antithrombotic use, and other neuroimaging markers of SVD, but it was not correlated with recurrent AIS or all-cause mortality. Furthermore, they stratified patients placed on antiplatelet therapy according to their CMB burden and concluded that patients with microbleeds had a similarly increased risk of ICH compared to those without microbleeds, and a high CMB number was associated with all-cause mortality but not vascular death. For warfarin users, the existence of CMBs did not increase the risk of either ICH or recurrent ischemic stroke, but as far as the location of CMBs was concerned, patients with mixed microbleeds had the highest 5-year absolute risk of an ICH. Thus, antithrombotic therapy should not be withheld from stroke patients with <5 microbleeds, as the risk of ICH is unlikely to outweigh the absolute risk of ischemic stroke recurrence. More research on the prognostic implications of CMB burden and their topographical distribution, as well as the possible underlying ethnic interactions, are needed in order to form valid clinical recommendations.

#### 4.2.4. Stroke Recurrence

Imaizumi et al. [71] enrolled noncardiogenic AIS patients and divided them into atherothrombotic infarction (ATI) and lacunar infarction (LI) groups to study MRI and U/S markers that could predict stroke recurrence and compare them between the two groups. Although both conditions are usually studied together as non-cardioembolic infarctions, they have different pathophysiologies, since ATIs originate from atherosclerosis, whereas LIs arise from hypertensive microangiopathy, so they can have different markers of stroke prognosis. Indeed, concerning ATI, all types of recurrent strokes were more frequent in patients with a mean intima-media thickness (IMT) greater than or equal to 1.1 mm, asymptomatic ICHs, and strictly lobar or mixed CMBs (deep and lobar) compared to those without them, but they found no difference between those without CMBs and those with strictly deep CMBs. On the other hand, LI patients with asymptomatic ICHs, asymptomatic LIs, severe WMHs, and strictly deep or mixed CMBs had a higher recurrence rate than those without, and notably, the incidences of those with mixed CMBs were greater than of those with strictly deep CMBs, which contradicted the ATI results, where the recurrence rate patients with mixed CMBs was lower than in those with strictly lobar CMBs. This study highlighted the importance of investigating the differences in AIS subtypes that can have prognostic implications based on their etiopathology and the authors called for further larger studies to validate the aforementioned findings.

Lau et al. [75] recruited patients from two cohorts, one comprising predominantly Caucasian patients with either TIA or ischemic stroke and one with predominantly Chinese patients with ischemic stroke, and studied the predictive ability of the total SVD score, which included lacunae, CMBs, WMH, and perivascular spaces. Participants with elevated total SVD scores had an increased risk of recurrent stroke, either ischemic or hemorrhagic—more particularly, they had an increased risk of both nondisabling and disabling recurrent ischemic strokes, but only with non-disabling ICH. Furthermore, the prognostic value of the total SVD score remained significant, regardless of the stroke subtype. Specifically for CMBs, the hazard of stroke recurrence grew with the CMB burden, especially when the patient had ≥5 microbleeds. These results validated the long-term prognostic utility of the total SVD score, which is robust to ethnicity and relevant to patients with either TIA or acute ischemic stroke.

Although CMBs are very common in the general elderly population and have been previously linked to stroke recurrence, research on the association of CMBs with TIA outcomes has been scarce, leading Fluri et al. [76] to investigate the impact of CMBs on patients experiencing TIA. Patients with CMBs had a greater risk of stroke within 3 months of the index event, compared with those without, especially when they also had acute ischemic lesions. However, the sample size of the study was too small to exclude the possibility of invalid findings or to establish an independent association between CMB burden and TIA prognosis, so the authors deemed the confirmation of their findings in larger population studies necessary.

#### 4.2.5. CMBs and Antithrombotic Therapy

Stroke patients with nonvalvular atrial fibrillation (NVAF) receive life-long anticoagulation therapy in order to be protected against recurrent and severe strokes. However, this intervention leads to an increased risk of hemorrhagic complications, which may be even worse in patients with multiple CMBs. To assess the risk of long-term mortality in cerebral infarction patients with NVAF, Song et al. [69] documented the presence, number, and topographical distribution of CMBs in the included participants and recorded their causes of mortality. After a median of 2.5 years follow-up, they concluded that the presence of CMBs significantly increased the mortality rate. Furthermore, both the number of CMBs and their location correlated with mortality, since ≥5 CMBs were an independent predictor of all-cause and AIS mortality, even after adjusting for age and sex, and a strictly lobar distribution increased the risk of death by hemorrhagic stroke, whereas mixed CMBs increased all-cause mortality. These results can be interpreted by the fact that lobar CMBs originate from amyloid angiopathy and CAA is an established cause of lobar ICH in aged patients on warfarin treatment. Thus, pretreatment MRI screening of the brain in aged stroke patients with AF could be beneficial for the prudent prescription of medication. To evaluate the benefit of antithrombotic therapy in contrast to the bleeding risk in patients with cardiogenic cerebral embolism caused by NVAF and CMBs, Wang et al. [67] also reported on cerebral hemorrhage occurrence and all-cause death. No connection between CMBs and the studied endpoints was established, even when the location and number of CMBs were also considered, but patients with CMBs in mixed regions and more than 10 CMBs were more likely to be put on anticoagulants. However, patients with CMBs and a history of cerebral hemorrhage had a significantly increased risk of all-cause death. In addition, patients with CMBs and hypertension or a history of CMBs exhibited an increased risk of cerebral hemorrhage occurrence. The authors concluded that there was no substantial evidence for antithrombotic therapy contraindication; hence, it should be used with caution, and blood pressure should be carefully controlled during antithrombotic therapy. Hert et al. [68] also studied the influence of SVD markers on the clinical courses and outcomes of stroke patients receiving anticoagulants for atrial fibrillation. Participants with CMBs had an increased risk of the composite endpoint—specifically, ischemic stroke, intracranial hemorrhage, and death—and notably, the risk of recurrent ischemic stroke was greater than that for ICH. The authors commented that the low frequency of ICH probably indicated that patients were not exposed to an extremely hazardous risk factor. However, CMB location was not related to the risk of composite outcomes and the presence of CMBs had no impact on disability. Furthermore, a threshold of CMB burden indicating an increased risk of recurrent stroke could not be established. On the other hand, WMHs were significantly related to composite outcomes, disability, and mortality. Finally, the authors concluded that stroke patients with atrial fibrillation should not be excluded from anticoagulation therapy based on the presence of CMBs alone, but rather, medication should be used with prudence and cerebrovascular risk factors should be controlled.

Lee et al. [59] attempted to reveal the risk factors of HT in patients who received anticoagulants early after a mild atrial fibrillation-related stroke, defined as infarction extending less than one third of the middle cerebral artery territory, one half of the anterior cerebral artery territory, or one half of the posterior cerebral artery territory and one half of one cerebellar hemisphere. They recorded each patient’s sex, history of hypertension, diabetes mellitus status, concomitant antiplatelet use, initial infarction volume, and cerebral microbleeds, but no correlation was established. Only posterior circulation infarction was a crucial independent risk factor of HT and none of the patients exhibited clinically significant symptomatic bleeding. The authors proposed that the posterior areas of the cerebral hemispheres may be particularly susceptible to hemorrhage due to the reduced density of sympathetic innervation in the posterior circulation, resulting in a decreased ability to resist excessive vasodilation. Likewise, Aoki et al. [60] reported on the effect of CMBs on mild non-cardioembolic ischemic stroke patients. After categorizing patients according to the number of CMBs recorded on admission, they noted that the different groups exhibited no difference in the rate of neurological deterioration, stroke recurrence, ICH, SAH, or extracranial hemorrhages within 14 days of symptom onset, nor did they display different risks of unfavorable outcomes at 3 months. Furthermore, since the administration of antiplatelet therapy is considered hazardous in patients with CMBs, they investigated whether the risk of ICH in patients treated with a combination of cilostazol and aspirin was increased compared to those treated with aspirin alone and found no alteration either in the efficacy or in the safety outcomes of the different groups, even when the location of the CMBs or the presence of ≥10 CMBs was taken into consideration. Although these findings cannot be generalized to all dual antiplatelet therapy regimens, the authors concluded that the combination of cilostazol and aspirin is safe for AIS patients, regardless of the CMB burden.

Finally, to assess the bleeding risk of stroke patients receiving antithrombotic therapy, Imaizumi et al. [72] recruited patients and categorized them according to the presence of CMBs and their medication history. As far as antiplatelet therapy was concerned, there was no correlation between deep CMBs and ICH occurrence, and this remained true when the number of CMBs was ≥3. However, when warfarin was concerned, although there was no difference in ICH events between patients with and without deep CMBs, the risk of hemorrhage was significantly higher for patients with ≥3 CMBs. The authors noted that the patient sample was too small to draw safe, clinically helpful conclusions, but they urged physicians to take caution when warfarin is used in patients with a great number of deep CMBs and suggested that the prescription of dabigatran is possibly preferable.

#### 4.2.6. Post-Stroke Depression, Cognitive Impairment, and Fatigue

Physical inactivity and cognitive impairment hamper active rehabilitation and the regaining of function after ischemic stroke, so their early recognition and restriction could be vital for a rapid recovery. Although WMHs have been previously linked to vascular depression, until a study was conducted by Tang et al. [66], no studies had focused on brain imaging markers as possible predictors of non-remission in post-stroke depression (PSD). To evaluate the impact of CMBs on PSD outcomes, Tang et al. [66] tested AIS patients with the 15-item Geriatric Depression Scale (GDS) 3 months after the index event and repeated the examination at 15 months for those who exhibited PSD, defined as a GDS score of ≥7. Patients were split into non-remitters if they remained depressed or remitters if they recovered. According to their results, lobar CMBs were associated with a lower remission rate of poststroke depression, but there was no correlation between baseline GDS scores and CMBs. In their sample, only a small percentage of patients received either pharmacological or psychological treatments; thus, the results reflected the effect of CMBs on the natural course of PSD. Further research on the impact of CMBs on the treatment outcomes of PSD and the possible contribution of CMBs to depression in the general elderly population is recommended.

Gregoire et al. [73] studied the correlation between CMBs and cognitive function in AIS and TIA patients. They documented CMBs, WMHs, lacunae, and territorial cortical infarcts on patients’ MRIs and examined them in regard to seven cognitive domains: current intellectual functioning, verbal and visual memory, naming skills, perceptual functions, speed and attention, and executive functions. CMBs were found to be more common only in patients with executive impairment, and the presence of at least one strictly lobar CMB more than doubled its likelihood. More than five strictly lobar CMBs were significantly linked to executive impairment, independently of age, WMC severity, and hypertension. Strictly lobar CMBs are a presumed marker for CAA, making them a possible risk factor for cognitive impairment and dementia, which might be preventable with an improved understanding of the underlying mechanisms contributing to their onset. Precise mapping of CMBs throughout the brain, with an assessment of damage to surrounding tissues, may be a helpful future approach for the diagnosis, treatment, and prevention of cognitive impairment in stroke populations. Brundel et al. [74] also investigated the relationship between CMBs and the long-term cognitive performance of minor stroke (mRS ≤ 3) or TIA survivors who received prophylactically anticoagulants or antiplatelet agents. They rated CMBs, atrophy, lacunae, and WMH on patients’ MRIs and assessed their cognitive status every 6 months for 4 years to uncover any decline. Microbleeds were not associated with long-term cognition, even when patients with multiple microbleeds (≥2) were compared to those without microbleeds. In contrast, the presence of lacunae and subcortical atrophies negatively influenced cognitive performance. Even though previous researchers have noted associations between cerebral microbleeds and cognitive performance deficits, they were restricted to executive functioning or appeared only in patients with lobar or multiple microbleeds. Hence, the authors concluded that unlike other signs of SVD, CMBs do not have a clinically meaningful relationship with cognition in the long run in the general stroke population.

Further on the quality of life of stroke survivors, Tang et al. [61] attempted to uncover the link between CMBs and post-stroke fatigue (PSF), which is a common and disabling problem. Assessing AIS patients with the Fatigue Severity Scale 3 months after the index event, they noted that although CMBs were not increased in the PSF group in a statistically significant manner, the presence of deep CMBs was an independent predictor of PSF. These findings could be explained by the fact that deep CMBs are linked to hypertensive vasculopathy, since hypertension has been previously reported as a risk factor for PSF. 

Taking all these results into consideration, it becomes clear that medication therapy and management of AIS patients require a careful individualized risk-to-benefit ratio evaluation. Although studies have yielded inconclusive conclusions regarding the effect of CMBs on stroke prognosis, the aforementioned findings have expanded our current understanding of stroke pathophysiology and enriched our knowledge about the underlying mechanisms linking the CMB distribution to varying stroke outcomes. Further research on patients with an increased burden of CMBs is needed for the continuous improvement of stroke survivors’ treatment and thus their quality of life.

### 4.3. Hemorrhagic Stroke

#### 4.3.1. Intracerebral Hemorrhage

Although the role of hypertensive arteriopathy and cerebral amyloid angiopathy in ICH etiopathology is well established, the possible impact of CMBs on hematoma volume and growth has not been widely studied. To address this gap, Shoamanesh et al. [77] investigated the potential influence of CMBs on 3-month functional outcomes and hematoma expansion within 24 h of symptom onset in ICH patients, along with the interaction of CMBs with intensive blood pressure treatment. To be eligible for their study, participants had to undergo CT on admission to validate that ICH volumes were less than 60 mL, as well as undergoing MRI T2*-GRE sequences to assess their burden of CMBs. In addition, they were required to have a GCS score ≥ 5 and an acute hypertensive response to be assigned to either standard treatment, targeting a BP reading between 140 to 179 mm Hg, or intensive treatment, targeting a BP reading between 110 to 139 mm Hg. The risk of death or disability or a hematoma expansion of at least one-third of the original hemorrhage volume did not seem to be influenced by the presence of CMBs, even when their number and location were taken into consideration. Moreover, the presence of CMBs did not alter the risk of complications, regardless of the treatment used. However, CMBs were linked to the presence of WMHs on MRI and renal dysfunction, which was expected since both CMBs and renal dysfunction are markers of hypertensive end-organ damage. However, the findings of the study were unforeseen, since previous research had demonstrated an association between CMBs and hematoma expansion in CAA-related ICH. To interpret their results, the authors hypothesized that patients with CAA-related ICH usually do not exhibit acute hypertensive responses and so were probably underrepresented in the trial.

Similarly, Warrier et al. [78] assessed the relationship of SVD markers, specifically WMHs, CMBs, and cortical siderosis, with hematoma volume, a hematoma expansion of at least one-third of the original hemorrhagic volume or at least 6 mL, and with the 3-month functional outcomes of patients. The presence of >3 CMBs on MRI predicted a poor outcome but had no impact on hematoma expansion and did not forecast a volume bleed greater than 30 mL. Of the markers examined, only high-grade WMH was able to predict the tree endpoints studied independently. These findings support the inclusion of WMHs in the current clinical prediction models as a selection tool for clinical trials of hemostatic therapy in ICH, which is important since poor patient selection is a major cause for trial failure, but the role of the other markers of SVD is still undetermined.

For long-term ICH prognosis, Miki et al. [79] tried to uncover the potential risk factors of ICH recurrence. They recorded each patient’s sex, age, and medical history, including hypertension, hyperlipidemia, diabetes mellitus, coronary disease, hyperuricemia, a history of smoking, medication history (antiplatelet drugs and anticoagulants), and the presence of microbleeds on MR images. Patients with a single hemorrhagic episode had no CMBs; particularly, patients without CMBs had a 0.128-times lower incidence of recurrent ICH. The other aforementioned factors showed no correlation with ICH. Hence, the authors of the study suggested that physicians should consider the presence of CMBs when deciding on the therapeutic management of first-ever-ICH patients. For example, strict regulation of blood hypertension and the termination of antithrombotic therapy or appropriate tailoring of its dosage could prove beneficial for patients with CMBs, but further research is needed to establish better guidelines for their management.

Furthermore, for chronic prognostication of ICH patients, Xu et al. [80] reported the effect of SVD burden, consisting of WMH, CMBs, lacunae, and EPVS, on functional outcomes, stroke recurrence, and mortality over a period of 5 years. Participants with ≥10 CMBs, higher periventricular WMH scores, or greater total SVD scores more often had poor functional outcomes. Stroke recurrence was more frequent in cases of periventricular WMH with higher total SVD scores, and in the case of lobar-located CMBs. Lastly, a lower survival rate was more common in patients with total SVD scores ≥ 2 or when there were ≥ 10 CMBs. Notably, of all SVD markers studied, only the presence of ≥ 10 CMBs was significantly related to death after adjustment for age, sex, hypertension, cardiac disease, GCS, albumin, and any complication. These findings support the utilization of MRI for timely long-term prognostication for primary ICH patients since the combination of ≥10 CMBs and periventricular WMHs has a detrimental effect on patients’ outcomes. Moreover, Pasi et al. [81] conducted a study of risk factors for functional decline, defined as an mRS score conversion from 0–3 to 4–5, in patients with a favorable outcome 6 months after ICH, whom they followed up for a median period of 9 years. Of the baseline characteristics, age, diabetes mellitus, ICH volume, and higher mRS scores at 6 months were independently associated with functional decline. Furthermore, the occurrence of a new clinical event, such as dementia, recurrent ICH, or AIS, significantly predicted functional decline. Across the markers of SVD studied, more specifically WMHs, CMBs, and cortical superficial siderosis (CSS), only strictly lobar and mixed CMBs was correlated with the occurrence of functional decline, indicating that CAA might be a potential contributor to functional decline. Taking all these findings into consideration, the authors highlighted the importance of assessing SVD markers upon baseline MRI of ICH patients for chronic functional outcome prognosis and suggested the vigilant monitoring of patients for the early recognition and management of dementia, as well as extra care for the prevention of thromboembolic events occurring after ICH.

#### 4.3.2. Subarachnoid Hemorrhage

With regards to SAH, Jeon et al. [82] investigated CMBs’ prevalence, radiological patterns, and impacts on patient outcomes. Almost half of the patients in the studied sample had CMBs and they were most often lobar in location, followed by deep and then infratentorial CMBs. Although patients with CMBs had greater mRS and lower TICS scores at discharge and at 3 months compared to those without CMBs, the relationship did not reach statistical significance. In contrast, CMBs were related to the presence of diffusion-weighted imaging lesions, even after adjusting for age and pre-existing hypertension. In this study, CMBs were very common among patients with SAH, raising the question of their potential influence on prognosis and treatment. Due to the small sample size, the natural history of CMBs in SAH survivors and their possible contribution to neurological complications such as cognitive impairment, depression, and gait instability could not be studied reliably.

Taking into account the aforementioned findings, it becomes clear that the stratification of patients with ICH who are more prone to hematoma expansion, poor functional outcomes, or stroke recurrence would have a clinically meaningful impact on the planning of care and preventive strategies to reduce the disease’s disabling burden. The presence of CMBs could indicate patients at higher risk of bleeding complications or long-term functional decline, emerging as a useful tool aiding timely and accurate therapeutic interventions tailored to each individual’s needs. Further research on the longitudinal association between baseline SVD burden, both individually and collectively, and long-term ICH prognosis would offer better insights into the pathophysiological mechanisms linking the two conditions and assist the revision of current guidelines to improve survivors’ quality of life.

## 5. Current Guidelines

In 2021, a review on the off-label use of IVT for AIS was published [83]. Although the authors reported that IVT in patients with extensive CMB burdens resulted in an increased rate of sICH and mortality, they noted that the probability of harboring > 10 CMBs was too low to justify the delay of therapy for pretreatment MRI acquisition to assess CMB count. Soon after this, the European Stroke Organisation (ESO) guidelines on IVT for AIS patients were issued [84], stating that patients eligible for IVT presenting within a 4.5-h time window should receive alteplase, even when the CMB number is unknown or if it has been previously established to be <10. Since pretreatment systemic screening with MRI to assess CMB burden could only be justified if it delayed therapy by less than 10 min, it is inadvisable for the general stroke population. However, if the CMB burden of a patient has been previously reported to be >10, IVT is contraindicated as the risk of hemorrhagic complications outweighs the possible beneficial effect of IVT. Moreover, the ESO has developed guidelines on antithrombotic treatment for the secondary prevention of stroke and other thromboembolic events in patients with stroke or TIA and NVAF [85], but unfortunately, no recommendations could be made regarding whether antiplatelet therapy is preferable over no antithrombotic treatment for patients with SVD, and the absence of CMBs was clearly stated to be an inadequate indicator of whether a patient should be treated with novel oral anticoagulants or vitamin K antagonists. Although for NVAF patients who have previously experienced an ICH of a CAA etiology oral anticoagulation attenuated the risk of death, no recommendations regarding anticoagulation resumption were made. Finally, the most recent guidelines for the management of patients with spontaneous intracerebral hemorrhage were issued by the American Heart Association/American Stroke Association (AHA/ASA) [86], which added a new section on neuroimaging findings suggestive of a hemorrhage-prone microvasculopathy in healthy individuals. In addition to being a helpful tool for recurrent ICH risk stratification, as a marker of underlying amyloid angiopathy, lobar CMBs can be also used in the estimation of first-ever-ICH risk in individuals with previous MRIs for other indications, thus aiding the planning of potential preventive treatments such as antithrombotic therapy or blood pressure lowering and optimal overall vascular management. Hence, we advise that in future ICH prognostication studies, SVD markers should be utilized as criteria for patient selection and risk stratification.

## 6. Study Limitations

Our systematic review is not without limitations. First, we cannot exclude the possibility that different studies published by the same research groups reported on data acquired from the same cohorts. Second, in the majority of the studies, patients experienced strokes that were of mild–moderate severity, since patients who were severely debilitated or deemed unfit for an MRI scan and demented patients who were unable to give consent were usually excluded, thus limiting the generalizability of the findings to the whole stroke population. Third, the effect of sociodemographic characteristics on the relationship of CMBs and patients’ functional outcomes has not been thoroughly studied. For this reason, we provided an extensive presentation of patients’ sociodemographic and clinical data, which might enable an indirect estimation of their role in an acute stroke setting and guide the design of future studies.

## 7. Conclusions

Taking all the abovementioned findings into consideration, in the present review we have provided an overview of the utility of assessments of preexisting CMBs as a prognostic biomarker of stroke patients’ recovery and the potential development of therapy complications. In spite of the lack of consensus in the literature, our findings support the evaluation of the CMB burden as a useful prognostic factor in stroke and reperfusion therapy, providing clinicians with crucial information for better counseling of patients and their families, a more appropriate selection of medical therapy, and a more accurate choice of patients for reperfusion therapy, according to the potential benefits they might experience. By assessing the CMB burden, it seems possible to separate patients with an unfavorable prognosis among all stroke patients from early follow-up to chronic follow-up and potentially to spot those patients who are more prone to hemorrhagic transformation, as patients with previous brain lesions are less able to compensate effectively for functional losses. Given that the assessment of the number and location of CMBs can provide valuable insights into stroke patients’ functional outcomes beyond clinical or other neuroimaging variables of SVD, it could significantly enhance previously established prognostic scores and advance overall stroke care. Additional studies on the association between the presence of multiple CMBs and the tendency for hemorrhagic complications after recanalization procedures are recommended in order to contribute important information for the formation of guidelines for the best management of stroke patients in several settings based on the CMB burden.

## Figures and Tables

**Table 1:** Characteristics of the 41 included studies.

Authors, Year of Publication	Type of Study	Number of Participants/ Mean Age	Demographics: Gender (M/F), Education (Years), BMI	Cerebrovascular Risk Factors (*n*)	Medication (*n*)	Previous Stroke (*n*)	Follow-Up Time	Method of CMB Assessment	Time of MRI Imaging/ Time to Recanalization	Scale of Stroke Severity and Prognosis/ Clinical Outcome	Main Results
**I. Reperfusion Therapy**											
***A. Intravenous Thrombolysis***											
Zand et al., 2017 [42]	Longitudinal	672/ 62 ± 14	350M/322F	Hypertension (518), diabetes mellitus (229), hyperlipidemia (215), atrial fibrillation (67), tobacco use (255)	-	181	Until discharge	Number and topographical distribution (strictly lobar or deep/infratentorial)	Pretreatment or within 24 h of treatment	NIHSS at admission, mRS at discharge	CMB presence did not invoke sICH more frequently after IVT, but CMB burden (>10 CMBs) had a statistically significant impact on sICH occurrence.
2. Nagaraja et al., 2018 [43]	Longitudinal	366/ 67 ± 15	199M/167F	Hypertension (216), diabetes mellitus (83), hyperlipidemia (146), atrial fibrillation (59), smoking (98), alcohol (109), peripheral vascular disease (10), myocardial infarction (15), coronary artery disease (57), congestive heart failure (12), valvular heart disease (8)	Antiplatelet (165), anticoagulant (18)	67	Until discharge	Brain Observer Microbleed Scale	On admission	NIHSS at baseline	Presence of CMBs significantly increased the risk for HT in AIS patients receiving IVT, regardless of lobar or deep location.
3. Schlemm et al., 2022 [44]	Longitudinal	459/ 68 (IQR 59–74)	288M/171F	Hypertension (242), diabetes mellitus (73), hypercholesterolemia (161), atrial fibrillation (50)	Platelet aggregation inhibitors (137), statins (137)	58	90 days	Number and topographical distribution (lobar, deep, or infratentorial)	At baseline/3.1 h (IQR 2.5–3.8)	NIHSS at baseline, mRS at day 90	The presence of CMBs had no impact on the 90-day functional outcome of AIS receiving IVT and although sICH was more common in patients with CMBs, the relationship failed to reach statistical significance.
4. Chacon-Portillo et al., 2018 [45]	Longitudinal	292/ 63 ± 15	141M/151F	Hypertension (230), diabetes mellitus (77), tobacco (85), alcohol (70), atrial fibrillation (58), hyperlipidemia (149)	-	63	90 days	Microbleed Anatomical Rating Scale	On admission	NIHSS at admission, at discharge, and day 90, mRS at discharge and day 90	Presence or location of CMBs did not influence ICH occurrence in AIS patients under thrombolysis, but the number >10 did.
5. Yan et al., 2015 [46]	Longitudinal	333	223M/110F	Hypertension (227), diabetes mellitus (67), atrial fibrillation (124), hyperlipidemia (159)	Previous use of aspirin or warfarin (45)	-	3 months	Number and topographical distribution (lobar, deep, or infratentorial)	Pretreatment/234 ± 92 min	NIHSS at baseline, mRS at 3 months	Patients with ≥3 CMBs had a significantly increased risk of PH and poor functional outcomes after IVT.
6. Capuana et al., 2021 [47]	Longitudinal	434/ 68.3 ± 13.5	264M/170F	Hypertension (285), diabetes mellitus (74), atrial fibrillation (70), hypercholesterolemia (138), smoking (146), congestive heart failure (10)	Antiplatelet (137), oral anticoagulation (16)	60	3 months	Number, size, and topographical distribution (lobar, deep, or infratentorial)	Pretreatment or within 24h of treatment/180 (IQR 135.0–222.25) min	NIHSS at baseline, mRS at 3 months	AIS patients receiving IVT were more likely to develop ICH, especially severe if they had CMBs, but no correlation between CMBs and poor functional outcomes or mortality was found.
7. Turc et al., 2015 [48]	Longitudinal	717/ 74 (IQR 60–83)	351M/366F	Hypertension (452), diabetes mellitus (113), atrial fibrillation (166), smoking (128)	-	71	3 months	Number and topographical distribution (lobar, deep, or infratentorial)	Pretreatment/ 152 (IQR 120–195) min	NIHSS at baseline, mRS at 3 months	CMB burden on pre-IVT MRI was not associated with sICH and with poor functional outcomes, after adjusting for age, hypertension, and atrial fibrillation, even when CMB location and presumed underlying vasculopathy was considered.
8. Dannenberg et al., 2014 [49]	Longitudinal	326/ 76 (IQR 68–84)	159M/167F	Hypertension (277), diabetes mellitus (74), atrial fibrillation (128), hyperlipidemia (171)	Statin (73), Antiplatelet (149), oral anticoagulation (5)	80	3 months	Number	Pretreatment/within 4.5 h of symptom onset	NIHSS at baseline, mRS at 3 months	With a growing number of CMBs, the rate of ICH and PH was increasing.
9. Drelon et al., 2020 [50]	Longitudinal	944	-	-	-	-	3 months	Number and topographical distribution (lobar or deep)	At admission/with a time window of 4.5 h	NIHSS at baseline, mRS at 3 months	Even though patients with r-ICH had more often strictly lobar CMBs and more in number; r-ICHs were better predicted by clinical variables.
10. Prats-Sánchez et al., 2016 [51]	Longitudinal	992/ 74.0 ± 12.6	525M/467F	Hypertension (718), diabetes mellitus (239), atrial fibrillation (239)	Antiplatelets (372), anticoagulation (67), statins (352)	142	3 months	Number and topographical distribution (lobar, deep, or mixed)	Within the first 14 days of the onset of stroke/within the first 4.5 h of symptom onset	NIHSS at baseline, mRS at 3 months	Patients with strictly lobar CMBs or RSI had a significantly increased risk for rPH.
***B. Mechanical Thrombectomy***											
11. Shi et al., 2016 [52]	Longitudinal	206/ 66.8 ± 17.6	87M/119F	Hypertension (135), diabetes mellitus (43), atrial fibrillation (82), dyslipidemia (64), coronary artery disease (43)	Aspirin (55), clopidogrel (13), warfarin (30), statins (59), antihypertensive drugs (103)	34	Until discharge	Microbleed Anatomic Rating Scale	Pretreatment	NIHSS at baseline, mRS at discharge	The number and topographical distribution of CMBs did not affect HT, functional outcomes, or in-hospital mortality of patients undergoing MT.
12. Lee et al., 2022 [53]	Longitudinal	577/ 67 ± 13	322M/255F	Hypertension (359), diabetes mellitus (159), atrial fibrillation (285), dyslipidemia (174), smoking (135)	-	102	3 months	Number	Pretreatment/within 24 h of symptom onset	NIHSS at baseline, mRS at 3 months	An increasing number of CMBs increases the rate of hemorrhagic complications and poor functional outcomes, partially mediated by WMH, HT, and lower successful reperfusion rates, listed in order of total CMB influence proportion.
***C. Any Reperfusion Therapy***											
13. Choi et al., 2019 [54]	Longitudinal	1532	855M/677F	Hypertension (1034), diabetes mellitus (438), atrial fibrillation (629), smoking (508)	-	208	3 months	Number and topographical distribution (lobar or deep/infratentorial)	On admission/Within 6 h of symptom onset	NIHSS at baseline, mRS at 3 months	Although there was no correlation between CMB presence and poor functional outcomes, in patients achieving recanalization, high CMB burden and lobar distribution were significantly associated with unfavorable functional outcomes. Furthermore, hemorrhagic complications were more common in participants with CMBs.
14. Gratz et al., 2014 [55]	Longitudinal	392 (174 IVT, 150 ET, and 68 both)/68.1 ± 13.7	223M/169F	Hypertension (249), diabetes mellitus (66), atrial fibrillation (142), smoking (167), hypercholesterolemia (213)	Antithrombotic therapy (173)	41	3 months	Microbleed Anatomic Rating Scale	Pretreatment	NIHSS at baseline, mRS at 3 months	The burden and location of CMBs were not associated with ICH occurrence, functional outcome, or survival rate. A higher CMB burden slightly increased the risk of ICH outside of the infarct.
**II. Ischemic Stroke**											
***A. Acute Ischemic Stroke***											
Takahashi et al., 2013 [56]	Longitudinal	187/ 74 ± 11	112M/75F	-	Antiplatelet (51), anticoagulant (136)	-	2 days	Number	At admission	NIHSS at baseline	CMBs did not suggest the occurrence of HT in patients who were on antithrombotic therapy.
2. Zand et al., 2018 [57]	Longitudinal	772/ 61.9 ± 14.2	398M/374F	Hypertension (601), diabetes mellitus (258), hyperlipidemia (250), smoking (284), atrial fibrillation (195)	NM	195	Until discharge	Number	NM	NIHSS at baseline	The presence of CMBs was not associated with in-hospital mortality of AIS patients, but if their number was ≥4, CMBs significantly increased the possibility of death.
3. Liu et al., 2015 [58]	Longitudinal	87/ 67.29 ± 12.45	49M/38F	NM	NM	NM	2 weeks	Presence	Within 24 h after symptom onset	-	Among the MR imaging features, CMBs, relative apparent diffusion, and venous anomalies were independent risk factors for HT of acute cerebral infarction.
4. Lee et al., 2018 [59]	Longitudinal	183/ 70.4 ± 10.4	107M/76F	Hypertension (34), hyperlipidemia (34), diabetes mellitus (117)	Warfarin (88), rivaroxaban (95), single antiplatelet (40), dual antiplatelet (5)	-	4 weeks	Presence	NM	-	Microbleeds did not correlate with hemorrhagic transformation in anticoagulated mild atrial fibrillation-related stroke patients.
5. Aoki et al., 2021 [60]	Longitudinal	1102/ 68 (IQR 60–77)	737M/365F	Hypertension (859), diabetes mellitus (258)	Cilostazol and aspirin therapy (439), aspirin (104)	123	3 months	Number	On admission	NIHSS at baseline, mRS at 3 months	The number of CMBs did not affect either the short-term or the 3 months clinical outcome. Furthermore, dual antiplatelet therapy with cilostazol and aspirin did not influence safety outcomes compared to aspirin alone, regardless of CMB burden.
6. Tang et al., 2014 [61]	Longitudinal	199	140M/59F, education 5.6 ± 5.2 (PSF) vs. 5.5 ± 4.5 (non-PSF)	Hypertension (155)	-	58	3 months	Topographical distribution (lobar, deep, or posterior fossa groups)	Within 7 days of admission	NIHSS within 2 days of admission, MMSE, GDS, BI, and the Chinese version of the Fatigue Severity Scale at 3 months	The existence of deep CMBs was significantly associated with post-stroke fatigue.
7. Nam et al., 2021 [62]	Cross-sectional	179/ 67 ± 10	108M/79F	Hypertension (77), diabetes mellitus (55), dyslipidemia (76), smoking (61)	-	-	3 months	Number (absence, single, multiple)	Within 24 h of admission	NIHSS at baseline, mRS at 3 months	CMB number was not associated with either END or with a 3-month unfavorable outcomes.
8. Ryu et al., 2020 [63]	Longitudinal	477/ 66 ± 14	294M/183F	Hypertension (359), diabetes mellitus (182), hyperlipidemia (143), smoking (221), coronary artery disease (75)	-	99	3 months	Presence	NM	NIHSS at baseline, mRS at 3 months	Total SVD scores were significantly associated with functional outcomes at three months following ischemic stroke and CMB presence was independently associated with higher 3-month mRS scores.
9. Sakuta et al, 2021 [64]	Longitudinal	240 (NIHSS score < 4 on admission)/ 66 (IQR 57–76)	187M/53F, BMI 23.7 (IQR 21.7–26.0)	Hypertension (165), diabetes mellitus (74), dyslipidemia (126), smoking (61), ischemic heart disease (20), peripheral arterial disease (6)	Single antiplatelet agent (150), dual antiplatelet agent (90), edaravone combined (94)	43	90 days	Number	On admission	NIHSS at baseline, mRS at day 90	In minor non-cardiogenic stroke patients treated with antiplatelet therapy, a higher CMB number led more frequently to poorer functional outcomes.
10. Kim et al., 2014 [65]	Longitudinal	225/ 67.6 ± 13.7	123M/102F	Hypertension (141), diabetes mellitus (64), dyslipidemia (87), ischemic heart disease (19), atrial fibrillation (53)	Antiplatelet agents (48), anticoagulant agents (4), statin (12)	44	6 months	Presence and topographical distribution (lobar, deep, or infratentorial)	Within 24 h of admission	NIHSS and mRS at admission, 24 h after admission, discharge, and 6 months after admission	The presence of CMBs was associated with poor functional outcomes both at discharge and at 6 months, but after adjustment for confounding factors, only infratentorial CMBs were correlated with poor outcomes.
11. Tang et al., 2014 [66]	Longitudinal	135/ 65.7 ± 11.0	66M/69F, education 5.9 ± 4.6	Hypertension (95), diabetes mellitus (58), hyperlipidemia (67), smoking (84)	-	26	15 months	Topographical distribution (lobar, deep, and posterior fossa groups)	Within 7 days of admission	NIHSS and mRS within 2 days of admission, MMSE and LSNS at 3 months, GDS at 3 and 15 months	Stroke patients with lobar CMBs had a lower remission rate of post-stroke depression.
12. Wang et al., 2019 [67]	Longitudinal	232	155M/77F	Hypertension (153), diabetes mellitus (85), smoking (85), atrial fibrillation (232)	Warfarin (44), rivaroxaban (9), dabigatran (88), aspirin (55), clopidogrel (36)	-	Mean of 22.4 ± 13.4 months	Number and topographical distribution (in a brain lobe of the cortex or a subcortical region, in a deep brain tissue region, or both)	At admission	NIHSS at admission	No difference in the occurrence of cerebral hemorrhage events or all-cause death was established between patients taking antithrombotic therapy for cardiogenic cerebral embolism with or without CMBs.
13. Hert et al., 2020 [68]	Longitudinal	320/ 78.2 ± 9.2	170M/150F	Hypertension (241), diabetes mellitus (62), smoking (81), hypercholesterolemia (122), alcohol (78)	DOAC (216), DOAC/antiplatelet (18), VKA (61), VKA/antiplatelet (15), antiplatelet (5)	-	Median of 754 (IQR 708–828) days	Number and topographical distribution (superficial or deep)	NM	NIHSS at baseline	Stroke patients with CMBs on anticoagulants for atrial fibrillation were at a higher risk of the composite endpoint (ischemic stroke, intracranial hemorrhage, death), but not for disability.
14. Song et al., 2014 [69]	Longitudinal	504/ 70 ± 11	288M/216F	Hypertension (392), diabetes mellitus (124), smoking (65), congestive heart failure (79), metabolic syndrome (180), coronary artery disease (109), peripheral artery disease (27), aortic atheroma (31)	Antiplatelet (224), anticoagulant (122), lipid-lowering agents (102)	93	Median of 2.5 years	Number, topographical distribution (strictly lobar, strictly nonlobar, and mixed)	Within 3 days of admission	NIHSS at admission	CMB presence raised the mortality rate, the greatest CMB burden predicted independently all-cause and ischemic stroke mortality and CMBs with strictly lobar distribution increased the hazard of hemorrhagic stroke mortality.
15. Lau et al., 2017 [70]	Longitudinal	1003/ 69 ± 12	601M/402F	Hypertension (657), diabetes mellitus (284), hyperlipidemia (256), smoking (297), atrial fibrillation (130)	Antiplatelet (218), warfarin (20), NOAC (3)	-	Mean of 37 ± 20 months	Microbleed Anatomical Rating Scale	Median of 4 days	mRS at discharge	Having ≥5 microbleeds independently predicted ICH occurrence, but not AIS recurrence. Furthermore, increasing the microbleed burden raised the risk of both ICH and all-cause mortality in antiplatelet users.
16. Imaizumi et al., 2019 [71]	Longitudinal	362 ATI and 309 LI	ATI: 211M/151F, LI: 169M/140F	ATI: hypertension (236), diabetes mellitus (101), smoking (109), LI: hypertension (193), diabetes mellitus (95), smoking (83)	ATI: statin (75), anti-platelet drugs (278), warfarin or DOAC (17), LI: statin (70), anti-platelet drugs (287), warfarin or DOAC (18)	-	Mean of 41 ± 52 months for ATI, mean of 51 ± 33 months for LI	Microbleed Anatomical Rating Scale	On enrollment	-	Stroke recurrence rates were significantly elevated in ATI patients with lobar or mixed (deep and lobar) CMBs and LI patients with either deep or mixed CMBs.
17. Imaizumi et al., 2013 [72]	Longitudinal	807/ 69.8 ± 12.0	456M/351F	-	Cilostazol (142), aspirin (203), clopidogrel sulfate (92), ticlopidine hydrochloride (34), warfarin (not stated)	-	0.5 to 71 months (mean of 31.6 ± 22.2 months)	Microbleed Anatomical Rating Scale	On admission	-	ICH occurrence was not more frequent in stroke patients with CMBs on antithrombotic therapy.
***B. Acute Ischemic Stroke or Transient Ischemic Attack***											
18. Gregoire et al., 2013 [73]	Longitudinal	320	190M/130F, median education 10 years	Hypertension (238), diabetes mellitus (63)	Antithrombotic (125)	91	3 months	Microbleed Anatomic Rating Scale	NM	Stroop test, Word Fluency, Trail Making Test Part B, Weigl Color Form Sorting Task, and Modified Card Sorting Test	Of the cognitive domains studied, only executive impairment was related to CMBs.
19. Brundel et al., 2014 [74]	Longitudinal	397/ 65 ± 12	137M/260F	Hypertension (185), diabetes mellitus (40), hypercholesterolemia (144), smoking (86), history of a cardiovascular event (41)	Antiplatelet (70), anticoagulant (5)	-	4 years	Microbleed Anatomical Rating Scale	Within 3 months after the ischemic event	mRS at baseline, TICS, or IQCODE after a mean interval of 3.8 years	Microbleeds, unlike the other markers of SVD, did not influence the long-term cognitive performance of stroke patients.
20. Lau et al., 2017 [75]	Longitudinal	2002	1121M/881F	Hypertension (1203), diabetes mellitus (411), hyperlipidemia (630), smoking (812), atrial fibrillation (288)	-	341	120 months	Microbleed Anatomical Rating Scale	NM	mRS at 1 month after recurrence	Increasing total SVD scores were correlated with a higher risk of recurrent ischemic stroke and ICH. In particular, microbleeds, especially when there were ≥5, raised the risk of recurrent stroke and especially of ICH.
***C. Transient Ischemic Attack***											
21. Fluri et al., 2012 [76]	Longitudinal	176	107M/69F	Hypertension (126), diabetes mellitus (31), hypercholesterolemia (71), smoking (39), atrial fibrillation (123), coronary heart disease (35), family history of stroke (40)	-	-	3 months	Number and topographical distribution	Within 24 h of the event	-	AIS after TIA was more frequent in patients with CMBs.
**III. Hemorrhagic Stroke**											
***A. Intracerebral Hemorrhage***											
Shoamanesh et al., 2018 [77]	Longitudinal	167/ 61.9 ± 13.2	98M/69F	Hypertension (125), smoking (71), cocaine use (10), congestive heart failure (7), atrial fibrillation (2), diabetes (31), ischemic heart failure 94), hyperlipidemia (45), peripheral vascular disease (3)	-	26	3 months	Number and topographical distribution (strictly lobar, strictly deep, or mixed)	NM	GCS and NIHSS at baseline, mRS at 3 months	There was no association between CMBs and death or disability at 3 months or hematoma expansion within 24 h of symptom onset, regardless of intensive vs standard blood pressure lowering, even taking into consideration the number and location of CMBs.
2. Warrier et al., 2021 [78]	Longitudinal	60/ 54.08 ± 11.57	47M/13F	Hypertension (60), smoking (22), alcohol use (10), diabetes mellitus (6), tobacco use (18), dyslipidemia (12), coronary artery disease (12)	-	0	3 months	Number	Within 5 days	mRS at 3 months	The presence of >3 CMBs increased the risk of poor outcomes but it did not influence hematoma volume or expansion.
3. Miki et al., 2020 [79]	Longitudinal	317	158M/159F	Hypertension (279), hyperlipidemia (71), smoking (105), alcohol use (97), diabetes mellitus (48), coronary disease (33)	Antiplatelet (70), anticoagulant (27)	49	Mean period of 1663days	Number	NM	mRS at discharge	Patients without microbleeds had a reduced risk of ICH recurrence.
4. Xu et al., 2021 [80]	Longitudinal	153/ 61.0 ± 12.2	111M/42F	Hypertension (123), hyperlipidemia (8), smoking (46), alcohol use (28), diabetes mellitus (16), cardiac disease (10)	-	15	Median of 4.9 years	Number and topographical distribution	At baseline	GCS at admission, mRS at the end of the follow-up	Patients with ≥10 CMBs had an increased risk of poor functional outcomes and death and when CMBs were lobar, patients experienced more frequent stroke recurrence. Notably, of all SVD markers, only CMBs ≥10 remained significantly related to mortality after adjusting for age, sex, and hypertension.
5. Pasi et al., 2020 [81]	Longitudinal	174/ 64.6 ± 13.5	108M/66F	Hypertension (107), dyslipidemia (56), smoking (38), alcohol use (57), diabetes mellitus (26), atrial fibrillation (25)	Antiplatelet (38), anticoagulants (24), antihypertensive (99), Statins (39)	14	Median of 9 years	Brain ObserverMicrobleed Scale	NM	NIHSS at baseline, mRS at 6 months, and 1, 2, 3, 4.5, 6, 8, and 10 years after the index ICH	Across the SVD markers, only strictly lobar or mixed CMBs independently predicted functional decline in patients with a favorable functional outcomes 6 months after ICH.
***B. Subarachnoid Hemorrhage***											
6. Jeon et al., 2014 [82]	Longitudinal	39/ 56 (IQR 44-65)	11M/28F	Hypertension (10), smoking (15), alcohol use (3), diabetes mellitus (4)	Antithrombotic agents (6)	1	3 months	Microbleed Anatomic Rating Scale	Within 7 days	mRS and TICS at discharge and 3 months	Although patients with CMBs had higher mRS scores at discharge and 3 months, the relationship did not reach statistical significance.

## Data Availability

All data discussed within this manuscript are available on PubMed.

## Abbreviations

CMB: cerebral microbleed; NIHSS: National Institutes of Health Stroke Scale; MRI: Magnetic resonance imaging; T2*-GRE: T2*-weighed gradient-recalled echo; ICH: intracerebral hemorrhage; CAA: cerebral amyloid angiopathy; SVD: small vessel disease; WMH: white matter hyperintensity; MMSE: Mini-Mental State Examination; APOE: apolipoprotein E; MARS: Microbleed Anatomic Rating Scale; AIS: acute ischemic stroke; PRISMA: Preferred Reporting Items for Systematic Reviews and Meta-Analyses; BMI: body mass index; mRS: Modified Ranking scale; GDS: Geriatric Depression Scale; BI: Barthel index; LSNS: Lubben Social Network Scale; GCS: Glasgow Coma Scale; TICS: Telephone Interview for Cognitive Status; IQCODE: Informant Questionnaire on Cognitive Decline in the Elderly; ICH: intracerebral hemorrhage; sICH: symptomatic intracerebral hemorrhage; IVT: intravenous thrombolysis; HT: hemorrhagic transformation; IQR: interquartile range; PH: parenchymal hemorrhage; r-ICH: remote intracerebral hemorrhage; RSI: recent silent ischemia; rPH: remote parenchymal hemorrhage; MT: mechanical thrombectomy; ET: endovascular therapy; NM: not mentioned; PSF: post-stroke fatigue; END: early neurological deterioration; DOAC: direct oral anticoagulants; VKA: vitamin K antagonist; NOAC: novel oral anticoagulant; ATI: atherothrombotic infarction; LI: lacunar infarction; TIA: transient ischemic attack; rt-PA: recombinant tissue plasminogen activator; CSS: cortical superficial siderosis; sHT: symptomatic hemorrhagic transformation; rADC: relative apparent diffusion; SBI: silent brain infarct; PSD: poststroke depression; WMC: white matter changes; NVAF: nonvalvular atrial fibrillation; SAH: subarachnoid hemorrhage; BP: blood pressure; EPVS: enlarged perivascular space; ESO: European Stroke Organisation; AHA/ASA: American Heart Association/American Stroke Association.
